# Supplementary figures and images for: Opisthorchis viverrini, Clonorchis sinensis and Opisthorchis felineus liver flukes affect mammalian host microbiome in a species-specific manner
Source: PLoS Negl Trop Dis. 2023 Feb 13;17(2):e0011111. doi: 10.1371/journal.pntd.0011111 (PMC9956601; doi:10.1371/journal.pntd.0011111)

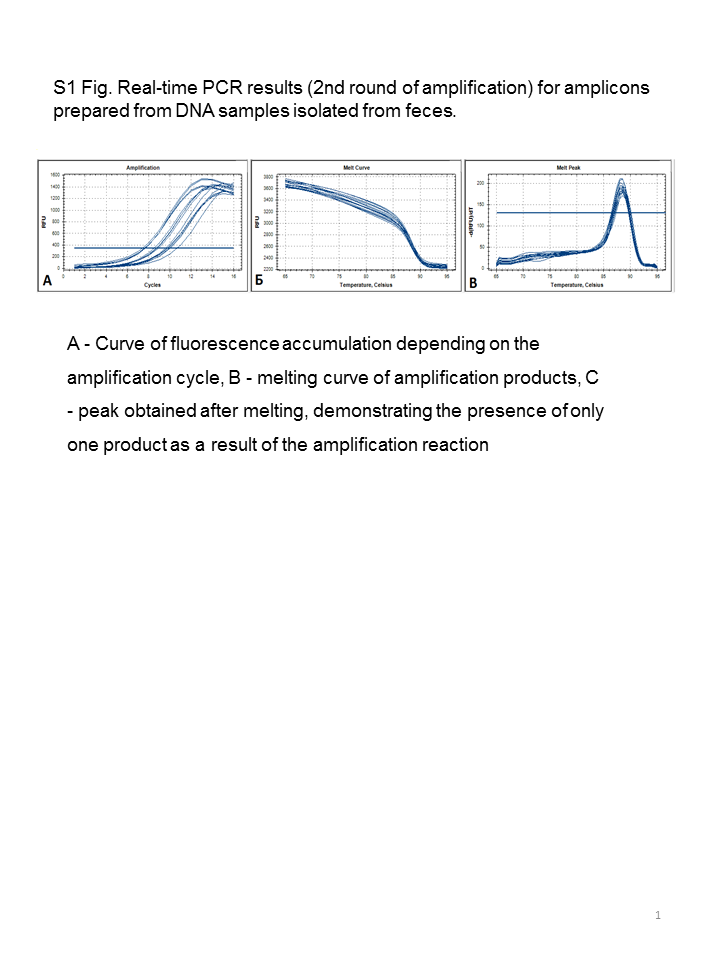

Supplement: S1 Fig — (TIF) [file pntd.0011111.s001.tif]

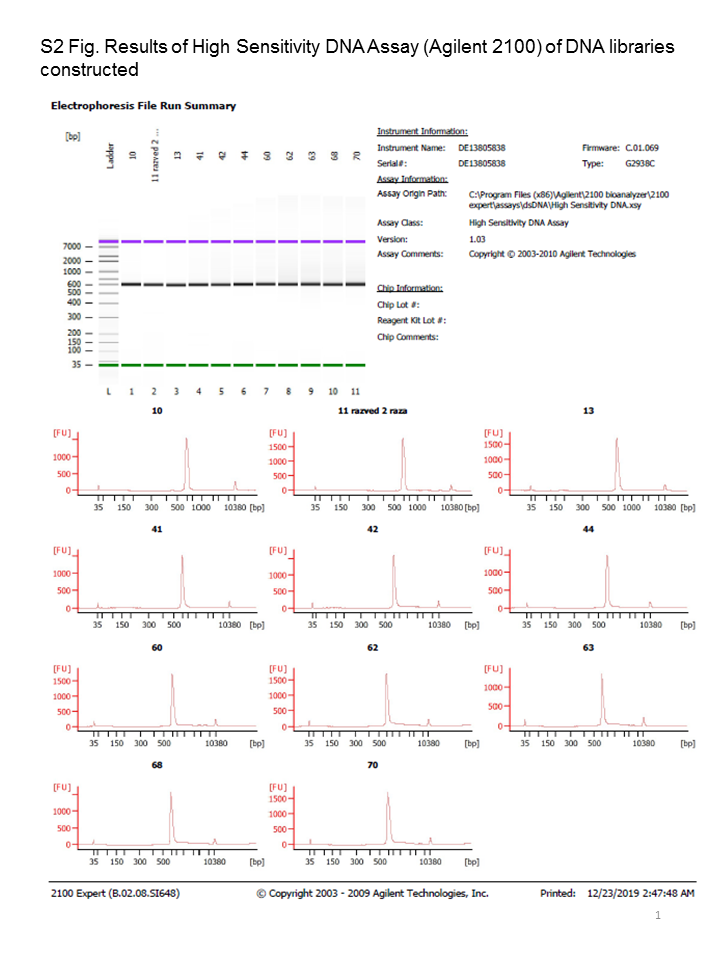

Supplement: S2 Fig — (TIF) [file pntd.0011111.s002.tif]

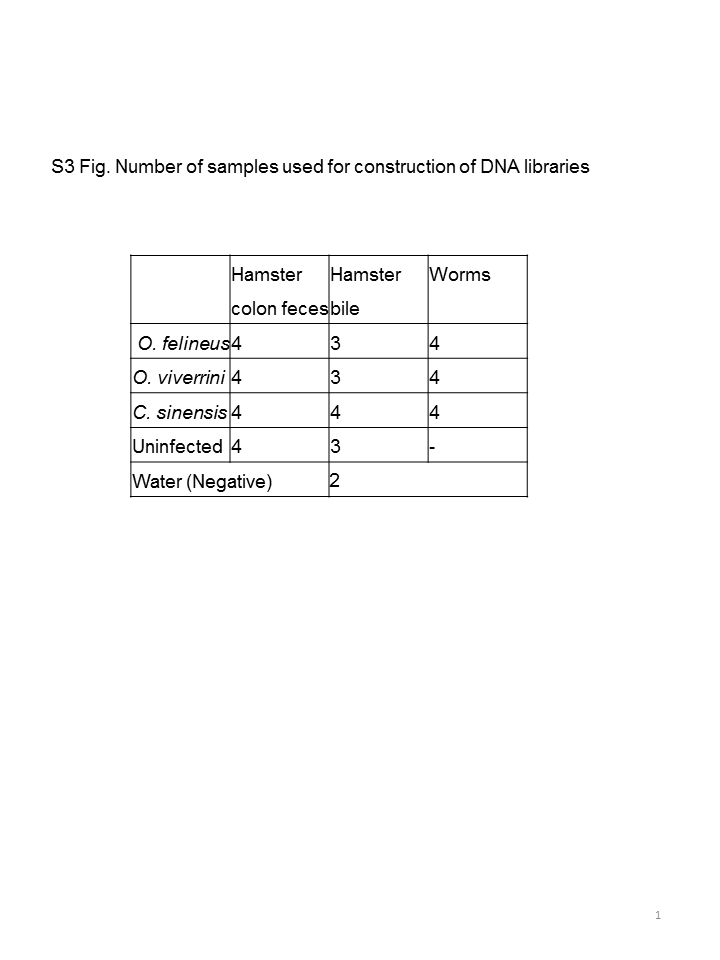

Supplement: S3 Fig — (TIF) [file pntd.0011111.s003.tif]

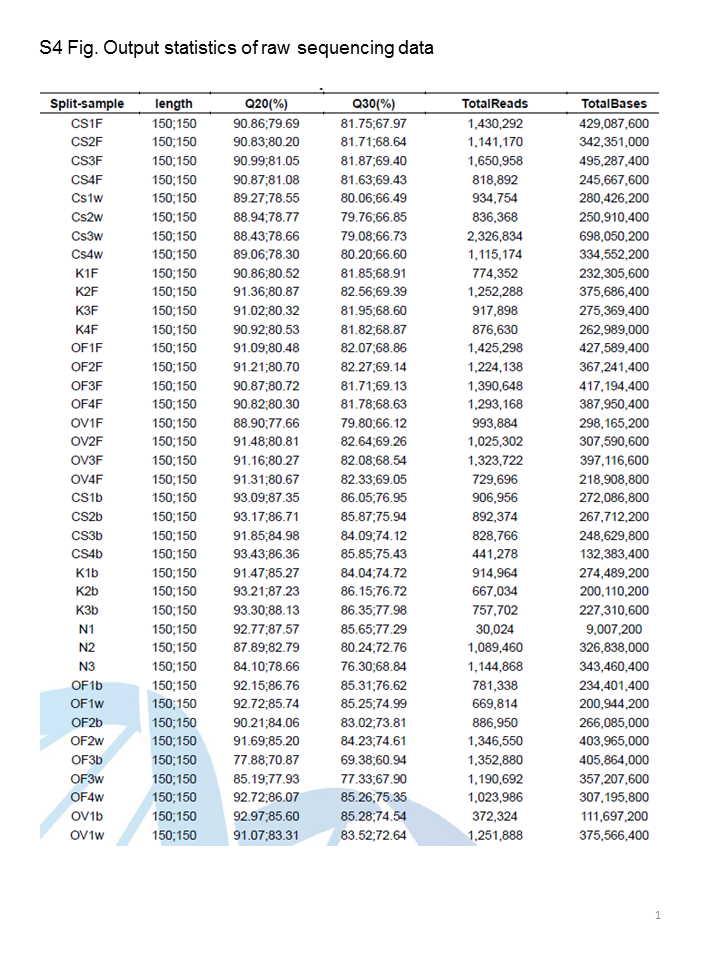

Supplement: S4 Fig — (TIF) [file pntd.0011111.s004.tif]

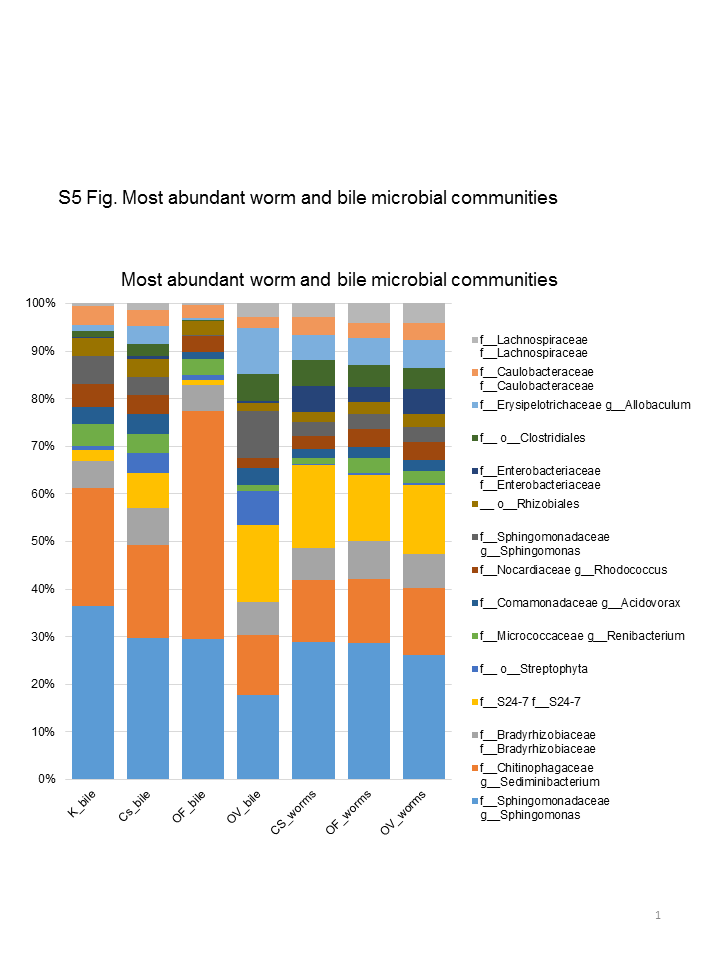

Supplement: S5 Fig — (TIF) [file pntd.0011111.s005.tif]

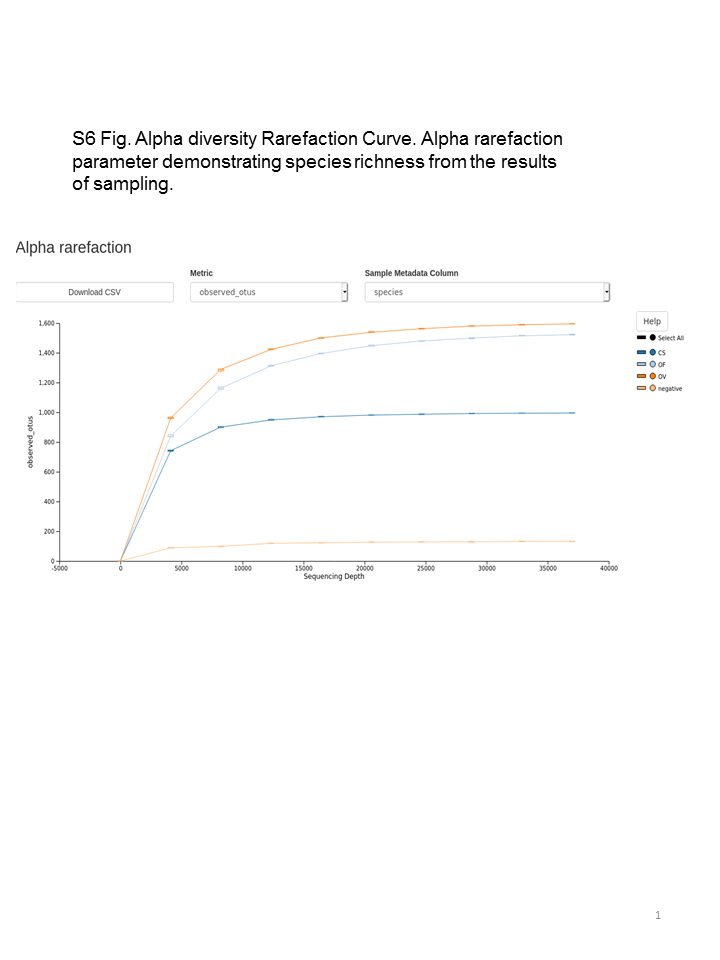

Supplement: S6 Fig — Alpha rarefaction parameter demonstrating species richness from the results of sampling. (TIF) [file pntd.0011111.s006.tif]
