# Supplementary material for: Opisthorchis viverrini, Clonorchis sinensis and Opisthorchis felineus liver flukes affect mammalian host microbiome in a species-specific manner
Source: PLoS Negl Trop Dis. 2023 Feb 13;17(2):e0011111. doi: 10.1371/journal.pntd.0011111 (PMC9956601; doi:10.1371/journal.pntd.0011111)
Supplement: Attachment — Submitted filename: Reviewer comments for PNTD-D-22-01019.docx [file pntd.0011111.s008.docx]

**PLOS Neglected Tropical Diseases [PNTD-D-22-01019]**

The manuscript entitled “Opisthorchis viverrini, Clonorchis sinensis and Opisthorchis felineus liver flukes affect mammalian host microbiome in a species-specific manner” by Pakharukova et al. showed that infections with these trematodes alter the composition of the microbiota in the bile and colon feces of experimental SPF animals and also examined the microbes of all liver flukes. There are several points concerning clarity in the manuscript. I think that the authors’ analysis needs to be reconsidered to make the most of the data they have collected. The author unclears to mention bioinformatics analysis in the method section, and the results are confusing. For example, OTU removal can have an effect on alpha diversity indices. Furthermore, how similar are the sequences assigned to the same OTUs, and what do you do for future annotation? The manuscript format must be improved for PNTD's style. I recommend a major revision.

**Abstract**

- Lines 40-46; The authors highlight O. viverrini and C. sinensis in the result section, but no mention of O. felineus or the characteristics of their result.

**Methods**

- Line 132; In the SPF animals, authors should define what is free, whether it is bacterial free, helminth free, or both.
- Line 147; As I understand it, the other group is the uninfected group, which should also be mentioned in it.
- Line 157; Please check the format of the PNTD guide for the authors.
- Lines 164-165; According to the sentence "Pieces (10 to 15 mg) of frozen samples of individual adult worms were subjected to DNA extraction" How did the author process this? Did the author cut the worm's body? Do you utilize the whole worm body? What section of the worm's body was analyzed? And do all of the samples come from the same section? Because the quantity and species of bacteria in each organ vary, the author should clarify.
- Lines 173; Incomplete references to the author's supplementary information. Please double-check this error, which can be found throughout the article.
- Lines 183; As for negative control samples, what type of water was used? The author should provide details.
- The BioProject PRJNA866652 is not available online may be a reviewer link needs to be given for review.

**Results**

- Line 251; Author's features = Operational Taxonomic Units (OTUs) should also be specified. I strongly suggest authors provide supporting data indicating the abundance of each OTU (number of sequences) in each library. The authors must recognize that in this case, OTU abundance must be used to determine alpha diversity. The removal of OTU before or after taxonomic analysis, as well as before or after alpha diversity, should be clearly explained by the author.
- Lines 262-264; …“For instance, alpha diversity shows the richness of phylogeny. Beta diversity is calculated as the number of species that are not the same in two different groups.”…. In my opinion, this sentence does not have to be included in the results.
- Line 266; This is incorrect information. Fig1B is not Faith’s PD. Please check again. This, and other mistakes, may be found throughout the article.
- Line 281; I recommend writing text in sequence with the figure or rearranging the figure. It is recommended to do the same for all of the author's figures.
- Lines 286-292; The author should rewrite and point to the findings that the author aims to demonstrate. Please check the information since, as indicated in Figure 2, the worm specimen contains 16 phyla.
- Line 295; Figure 2. The author is not shown at the genus level.
- Line 297; Fig 2C, please add Y-axis name.
- Line 307; Throughout the text, but especially in Fig 2C, it is unclear how the authors combined experimental data from individuals. For example, using an average abundance. The methodology is not described.
- Line 317; Figure 3, I don’t think that Figure 3 contributes much to description of data. Maybe an alternative version of this figure or just description of data in main text.
- Line 351; the word “new genera” What exactly does this mean? It should be made clear. How is this new?
- Throughout the manuscript, I recommend reordering your Figures and Supplementary tables. Let's start with the numbers, in the order they were mentioned.
- Line 388; The result in Fig.5, it seems do not reach the level of statistical significance. I recommend removing all reporting of non-significant results.
- Line 395; As I suggested, OTU removal can have an effect on alpha diversity indices but can do after taxonomy. However, how similar are the sequences that were allocated to the same OTUs, and what do you do for further annotation? It's not mentioned in the method section.
- Line 413; There is conflicting information with the mentioned above, that there are 50 or 54 families.
- Line 415; I recommend removing Fig. 6C since it seems less important. Maybe an alternative version of this figure, for instance, by adding a figure of relative abundance (lines 420–428).
- Line 465; This is incorrect information. Fig6A is not matched with the text. Please check.
- Line 506; The author has written too freely for this sub-topic. Because the author has provided information about the outcomes in each sub-topic, I can deal with the main point. This sub-topic should provide the most important results that the reader needs to know.
- Line 570; The letters pointing in the image should be reshaped, such as E to e, to make it easier to see. Moreover, I see a duplicate sentence in Fig8 legend, please check.

**Discussion**

- The authors have introduced the importance of liver flukes in the development of cholangiocarcinoma. The author's question about the mechanisms underlying helminth-induced carcinogenesis with their microbes. Therefore, the authors should discuss the results obtained in the context of the bacteria found in the worm and in the host bile duct and should also discuss why this bacterium is found and how it is important or transmitted in the development of cholangiocarcinoma. For example, the results in Fig2, which, if possible, should be reached at the genus or species level to identify the function and effect of that back. Which species is related to inflammation or fibrosis? This is useful in predicting pathogenesis in the host bile duct or trying to find out which microbiome data has such an organism in your results.
- Lines 706-708; Did you find *Helicobacter pylori* in this study?
