# Supplementary material for: Opisthorchis viverrini, Clonorchis sinensis and Opisthorchis felineus liver flukes affect mammalian host microbiome in a species-specific manner
Source: PLoS Negl Trop Dis. 2023 Feb 13;17(2):e0011111. doi: 10.1371/journal.pntd.0011111 (PMC9956601; doi:10.1371/journal.pntd.0011111)
Supplement: Attachment — Submitted filename: Responses_Reviewer comments_final.docx [file pntd.0011111.s009.docx]

**Responses to Reviewers’ comments:**

**General Response to Reviewer #1:**

Thank you for the review of our paper and for the comments, corrections, and suggestions. We believe the paper has been significantly improved. Our answers to your questions are provided below in blue font. We are also enclosing the file *manuscript_highlighted.doc:* the changes in the text of the article are shown as red text.

**Reviewer #1:**

*Methods*

1. The objective of this study is clearly address with clear hypothesis. However, in the method, due to the geographically different of each type of parasite, the authors did not provide several informations about the animal rearing process which might effect the microbiome such as location of hamster rearing, how the metacercaria was prepared and kept during the shipment if all group of hamsters was kept in the same location, or if the hamsters was kept in different locations, how the hamsters food was handling etc.

We apologize for the lack of clarity in the description of the study protocol. In fact, all the conditions for keeping hamsters were the same, because all manipulations regarding hamster rearing, infecting them with metacercariae, and further experiments were carried out at the same location by the same person in the SPF Animal Facility at the Institute of Cytology and Genetics, Novosibirsk. Accordingly, the experimental conditions were the same, in particular, the food handling and care of the hamsters.

Metacercariae were isolated in different geographical locations by different people, and delivered on ice. Differences in this study that might influence the outcomes of this comparative research were at the stage of fish processing and isolation of metacercariae. Although this preparation step was conducted according to a standard protocol, we cannot exclude variations at this step. We have added this to the text of the manuscript as a limitation of this study (lines 155-157).

*Results*

2. Most of the results and figures are clearly demonstrate. However, there are some sentence in the results part and discussion part which not agree with each other for example "No Helicobacter species were found" in Line 534 (Results) but in the discussion part the authors state that "On the other hand, no significant difference in Helicobacter abundance were observe..." (Line 706-707; discussion). The authors must carefully check about these points.

In the previous paper (Pakharukova et al., 2021) we showed the presence of Helicobacter bacteria after liver fluke infection, but in this study we did not show the presence of Helicobacter. Indeed, one may get the wrong impression why we got contradictory results about *Helicobacter pylori* presence. The differences between the previous study and this research are due to the methods we used. In particular, to detect the presence of Helicobacter bacteria, it is necessary to use gene-specific primers (as we did) for the *Helicobacter UreaA* gene and raised the number of cycles in the PCR to 40-45. This work on 16S microbiome research based on amplification of the 16S ribosomal RNA gene using non-specific bacterial primers. The best strategy for 16S microbiome is the maintaining as much bacterial diversity as possible, that is why the number of cycles in the PCR reaction was as minimal as possible. So, for bile we applied 25 cycles, for feces - 20 cycles, for the trematodes themselves - 10 cycles.

Unfortunately, minimal number of PCR cycles was not enough to identify all low-presented bacterial species. The results of this study suggest that Helicobacter pylori is not a major species after the infection with liver flukes. We supposed that, in particular it might be due to that fact, that research was conducted in SPF Animal Facility, so the animals initially were free from many specific pathogens.

To remove the lack of understanding we modified the text in the Discussion section.

3. For the result presentation, the authors should considers the revise by avoiding the use of 'g_' 'f_' but using 'genus' 'family' following the name instead.

Thank you, we revised the manuscript accordingly.

*Conclusions*

4. The authors should address more about the effect of the changes of each type of microbe on the pathology of biliary tract which currently less mentioning. Most of the discussion part is only compared the result with previous findings which not helpful with the understanding of the topic under study.

According to your suggestion, we significantly modified the Discussion section (lines 586-622) and Conclusion section (lines 734-746). We inserted the text about the potential mechanisms underlying helminth-induced carcinogenesis, we also discuss the results obtained in the context of the bacteria found in the host bile duct.

5. The authors still not discuss about limitations of the study.

We have added limitations to the text of the manuscript (lines 155-157).

*Summary and General Comments*

6. Overall, the findings from the study is novelty and displays an important piece of knowledge in the study field. However, several part still need to modified especially on the discussion which will improve the understanding of the topic under study.

According to your suggestion, we significantly modified the Discussion section (lines 586-620) and Conclusion section (lines 734-746).

**General Response to Reviewer #2:**

Thank you for the review of our paper and for the comments, corrections, and suggestions. We believe the paper has been significantly improved. Our answers to your questions are provided below in blue font. We are also enclosing the file *manuscript_highlighted.doc:* the changes in the text of the article are shown as red text.

**Reviewer #2:**

1. There are several points concerning clarity in the manuscript. I think that the authors’ analysis needs to be reconsidered to make the most of the data they have collected. The author is unclaer to mention bioinformatics analysis in the method section, and the results are confusing. For example, OTU removal can have an effect on alpha diversity indices.

We revised the ST2 Table. Now it represents the pipeline of implemented bioinformatics steps (step-by-step) for clear demonstration.

2. Furthermore, how similar are the sequences assigned to the same OTUs, and what do you do for future annotation?

DADA2 tool in QIIME2 protocol compares and keeps only those reads that have homologs in a certain database of bacterial sequences, i.e. compared with real bacterial sequences. DADA2 generates a feature Table with counts (more than 17 thousands). No clustering of sequences based on similarity was conducted, only 16S amplicon sequence variants were present.

3. The manuscript format must be improved for PNTD's style.

We revised the manuscript according to the PNTD's Instruction for authors

*Abstract*

4. - Lines 40-46; The authors highlight O. viverrini and C. sinensis in the result section, but no mention of O. felineus or the characteristics of their result.

We revised the Abstract

*Method*

5.- Line 132; In the SPF animals, authors should define what is free, whether it is bacterial free, helminth free, or both.

Every 3 months SPF hamsters are tested for the absence of specific viruses, bacteria, endo- and ectoparasites according to the “Recommendations for the health monitoring of mouse, rat, hamster, guinea pig and rabbit colonies in breeding and experimental units”. Recommendations of the European Laboratory Animal Science Associations 2014 (FELASA). The list of pathogens tested might be sent upon request.

6. - Line 147; As I understand it, the other group is the uninfected group, which should also be mentioned in it.

Corrected (lines 149)

7. - Line 157; Please check the format of the PNTD guide for the authors.

Corrected

8. - Lines 164-165; According to the sentence "Pieces (10 to 15 mg) of frozen samples of individual adult worms were subjected to DNA extraction" How did the author process this? Did the author cut the worm's body? Do you utilize the whole worm body? What section of the worm's body was analyzed? And do all of the samples come from the same section? Because the quantity and species of bacteria in each organ vary, the author should clarify.

We actually weighed the worms preliminary to roughly estimate a weight of a worm of each species, because they differ in size.

We did not utilize a particular part of the worms, but used a whole individual Clonorchis worm, or 2-4 O. viverrini and O. felineus worms, since they were smaller. In the previous version of the text, there was an incorrect expression, which actually meant that we cut them into small pieces before extracting DNA.

The text has been clarified now (lines 166-167).

9. - Lines 173; Incomplete references to the author's supplementary information. Please double-check this error, which can be found throughout the article.

Corrected

10. - Lines 183; As for negative control samples, what type of water was used? The author should provide details.

We used the Water Molecular Biology Grade, PanReac AppliChem. The details were inserted (lines 185-186).

11. - The BioProject PRJNA866652 is not available online may be a reviewer link needs to be given for review.

To assess the quality of the prepared and sequenced DNA-libraries, amplicon coverage and quality of first step filtering of raw data, we specifically provide table ST3 so that the reviewer is able to assess how many libraries were prepared and sequenced and judge the quality of the data.

The quality table (ST3) also clearly demonstrates the coverage, how many raw reads were originally per a sample, how many chimeric sequences were found during the filtering step, and how many reads per sample in went into following processing eventually.

Moreover, statistics of raw sequencing data is presented in SF5, main output statistics from Qiime2 is presented in SF6.

*Results*

12.- Line 251; Author's features = Operational Taxonomic Units (OTUs) should also be specified. I strongly suggest authors provide supporting data indicating the abundance of each OTU (number of sequences) in each library. The authors must recognize that in this case, OTU abundance must be used to determine alpha diversity. The removal of OTU before or after taxonomic analysis, as well as before or after alpha diversity, should be clearly explained by the author.

It is important, that “features” is not equal to “Operational Taxonomic Units (OTUs)”. DADA2 generates a feature Table with counts (no clustering of sequences based on similarity (OTUs), only 16S amplicon sequence variants). We specified the features as “amplicon sequence variants” in the text of M&M, lines 159

According to your suggestion we explained the filtering step in the manuscript (Table ST1 and M&M section, lines 259). Since filtering is a mandatory step in any protocol for 16S mass sequencing data analysis, we followed the QIIME2 protocol. The pipeline of implemented steps (step-by-step) is presented in ST1 of Suppl files. In accordance with the recommendations, the filtering was applied using the DADA2 tool, which keeps only those reads that have identical sequences in a certain database of bacterial sequences, i.e. compared with real bacterial sequences. Only after this step alpha and beta diversity was calculated, as well as taxonomic analysis was conducted (step-by-step pipeline is presented in ST1).

Removal of low-abundance features (1-2 counts) was applied after QIIME2 analysis but before the MetagenomeSeq analysis (as indicated in Suppl. ST1).

The feature Table contains more than 17 thousand lines and is not very informative and easy to read and understand, so we decided to omit it.

13. - Lines 262-264; …“For instance, alpha diversity shows the richness of phylogeny. Beta diversity is calculated as the number of species that are not the same in two different groups.”…. In my opinion, this sentence does not have to be included in the results.

Deleted

14. - Line 266; This is incorrect information. Fig1B is not Faith’s PD. Please check again. This, and other mistakes, may be found throughout the article.

Revised

15. - Line 281; I recommend writing text in sequence with the figure or rearranging the figure. It is recommended to do the same for all of the author's figures.

We rearranged the parts in the figure.

16. - Lines 286-292; The author should rewrite and point to the findings that the author aims to demonstrate. Please check the information since, as indicated in Figure 2, the worm specimen contains 16 phyla.

Figure 2B and the text were revised (lines 293-295)

17. - Line 295; Figure 2. The author is not shown at the genus level.

The legend was revised.

18. - Line 297; Fig 2C, please add Y-axis name.

Corrected

19. - Line 307; Throughout the text, but especially in Fig 2C, it is unclear how the authors combined experimental data from individuals. For example, using an average abundance. The methodology is not described.

The Figure 2C Legend was revised. We inserted the following sentence. “The average percent abundance is presented.”

20. - Line 317; Figure 3, I don’t think that Figure 3 contributes much to description of data. Maybe an alternative version of this figure or just description of data in main text.

We disagree with the comment, we believe that this is the only figure that, firstly, is illustrating the diversity at the class level, and secondly, shows the classes that appeared after the infection. In our opinion, it does not duplicate any other illustrative material and is important for understanding how rich the diversity is and how many classes change after infection. So, we would like to keep it.

21.- Line 351; the word “new genera” What exactly does this mean? It should be made clear. How is this new?

We replaced “new” by “infection-associated” (lines 355-356)

22. - Throughout the manuscript, I recommend reordering your Figures and Supplementary tables. Let's start with the numbers, in the order they were mentioned.

We reordered the Figures.

PNTD’s Instruction for Authors: “..We recommend that you cite supporting information in the manuscript text, but this is not a requirement. If you cite supporting information in the text, citations do not need to be in numerical order...”

According to the Instruction for Authors it is no need to rearrange the Supplementary Tables, so we would like to keep them as they are.

23. - Line 388; The result in Fig.5, it seems do not reach the level of statistical significance. I recommend removing all reporting of non-significant results.

We applied Kruskal Wallis test followed by post-hoc Dunn's test using Bonferroni correction to recalculate statistical significance. Now only significant results (padj < 0.05) are presented. We revised the Table ST17 and Figure 5 accordingly.

24. - Line 395; As I suggested, OTU removal can have an effect on alpha diversity indices but can do after taxonomy. However, how similar are the sequences that were allocated to the same OTUs, and what do you do for further annotation? It's not mentioned in the method section.

QIIME2 and DADA2 generates a feature Table with counts (more than 17 thousands). No clustering of sequences (OTUs Table construction) was conducted, only amplicon sequence variants were present. The pipeline of bioinformatics analysis is presented in ST1. Training Naive Bayes classifier and taxonomic Assignment was also conducted according to the QIIME2 https://docs.qiime2.org/2022.8/.

25.- Line 413; There is conflicting information with the mentioned above, that there are 50 or 54 families.

Revised

26. - Line 415; I recommend removing Fig. 6C since it seems less important. Maybe an alternative version of this figure, for instance, by adding a figure of relative abundance (lines 420–428).

We would like to keep this figure, because it illustrates the differences between uninfected and CS-infected feces. We rephrased the text of the results accordingly (lines 421-422).

27. - Line 465; This is incorrect information. Fig6A is not matched with the text. Please check.

Revised

28. - Line 506; The author has written too freely for this sub-topic. Because the author has provided information about the outcomes in each sub-topic, I can deal with the main point. This sub-topic should provide the most important results that the reader needs to know.

The sup-topic 3.6. was revised, only important results were kept.

29.- Line 570; The letters pointing in the image should be reshaped, such as E to e, to make it easier to see. Moreover, I see a duplicate sentence in Fig8 legend, please check.

The letters were reshaped in this figure and in all other figures. The Fig8 legend now does not contain any duplicate sentences.

*Conclusions*

30. The authors have introduced the importance of liver flukes in the development of cholangiocarcinoma. The author's question about the mechanisms underlying helminth-induced carcinogenesis with their microbes. Therefore, the authors should discuss the results obtained in the context of the bacteria found in the worm and in the host bile duct and should also discuss why this bacterium is found and how it is important or transmitted in the development of cholangiocarcinoma. For example, the results in Fig2, which, if possible, should be reached at the genus or species level to identify the function and effect of that back. Which species is related to inflammation or fibrosis? This is useful in predicting pathogenesis in the host bile duct or trying to find out which microbiome data has such an organism in your results.

According to your suggestion, we significantly modified the Discussion section (lines 586-622) and Conclusion section (lines 734-739). We inserted the text about the potential mechanisms underlying helminth-induced carcinogenesis, we also discuss the results obtained in the context of the bacteria found in the host bile duct.

31. - Lines 706-708; Did you find Helicobacter pylori in this study?

In this study we did not show the presence of Helicobacter.

To remove the lack of understanding, we have modified the text of the discussion section.

32. The author is unclear to mention bioinformatics analysis in the method section and the results are confusing. In particular, OTU removal can have an effect on alpha diversity indices. Furthermore, how similar are the sequences assigned to the same OTUs? Supporting data indicating the abundance of each OTU (number of sequences) in each library should be provided.

We revised the ST1 Table. Now it represents the pipeline of implemented bioinformatics steps (step-by-step) for clear demonstration. We modified the M&M section to refer to the Table ST1 and the bioinformatics pipeline, including filtering steps. Additionally, all filtering steps we explained (point 12, presented above).

The sequences assigned to the same feature were identical, no clustering (OTU) was applied. The filtering was applied using the DADA2 tool, which keeps real bacterial sequences. Only after this step alpha and beta diversity was calculated, as well as taxonomic analysis was conducted (step-by-step pipeline is presented in ST1).

OTU Table was not constructed, but a feature Table (amplicon sequence variant) was generated by DADA2 tool. The feature Table contains more than 17 thousand lines and is not very informative and easy to read and understand, so we decided omitting it. The details about DADA2 taxonomy assignment are described here https://benjjneb.github.io/dada2/index.html

**Responses to Reviewers’ comments:**

**General Response to Reviewer #4:**

Thank you for the review of our paper and for the comments, corrections, and suggestions. We believe the paper has been significantly improved. Our answers to your questions are provided below in blue font. We are also enclosing the file *manuscript_highlighted.doc:* the changes in the text of the article are shown as red text.

**Reviewer #4:**

1. Most results are the single description of the sequence results, i think it is better to add some comparison of the different microbiome of the three orgin.

According to your comment, we significantly modified the Discussion section (lines 586-622). We inserted the text about some comparisons of the host infected with different liver flukes.

2. The author have sequenced the composition of microbiota of bile and colon feces, the results is interesting, however, it have some different in the 3 kinds of parasite, so how we understanding ,it is better to described in the discussion.

We significantly modified the Discussion section (lines 586-622) and Conclusion section (lines 734-746). We inserted the text about the potential mechanisms underlying helminth-induced carcinogenesis, we also discuss the results obtained in the context of the bacteria found in the host bile duct.

3. Whether the author use hamster as the animal model need to be described in the manuscript.

Syrian hamster *Mesocricetus auratus* (Waterhouse, 1839) is a generally accepted model animal for studying opisthorchiasis caused by *O. felineus* and *O. viverrini* [Sripa et *al.*, 2012a; Mordvinov et *al.*, 2017b, 2021; Pakharukova et *al.*, 2018, 2019a,b,c, 2021; Arunsan et *al.*, 2019; Zaparina et *al.*, 2021]. These animals are susceptible to infection by liver flukes, which is not surprising because the main natural reservoir animal of *O. felineus* is the muskrat, of the Cricetidae family closely related to Syrian hamsters [Fedorov, 1979, 1989; Pakharukova and Mordvinov, 2022]. It must be noted that pathomorphological, histological, and biochemical characteristics of *opisthorchiasis* infection in hamsters (experimental opisthorchiasis) are similar to the manifestations of the disease in humans [Kovner et *al.*, 2019], including liver fluke–associated CCA. Sometimes mice and rats are used to study liver fluke infection, but liver fluke–associated CCA has not been obtained in these model [Uddin et *al.*, 2016; Rim, 2005].

We inserted the references in the M&M section (lines 131)
